# Differential effects of rice bran cultivars to limit *Salmonella* Typhimurium in chicken cecal *in vitro* incubations and impact on the cecal microbiome and metabolome

**DOI:** 10.1371/journal.pone.0185002

**Published:** 2017-09-22

**Authors:** Peter M. Rubinelli, Sun Ae Kim, Si Hong Park, Stephanie M. Roto, Nora Jean Nealon, Elizabeth P. Ryan, Steven C. Ricke

**Affiliations:** 1 Center for Food Safety and Department of Food Science, University of Arkansas, Fayetteville, Arkansas, United States of America; 2 Department of Food Science and Technology, Oregon State University, Corvallis, Oregon, United States of America; 3 Department of Environmental and Radiological Health Sciences, Colorado State University, Fort Collins, Colorado, United States of America; University of California Davis, UNITED STATES

## Abstract

In this study, rice brans from different cultivars (Calrose, Jasmine, and Red Wells) were assessed for their ability to inhibit *Salmonella enterica* serovar Typhimurium using an *in vitro* mixed anaerobic culture system containing cecal microbiota obtained from broilers of different ages. *Salmonella* Typhimurium was added to controls (feed only, cecal only, and feed + cecal material) and treatments (feed + cecal + different rice brans) and *S*. Typhimurium populations were enumerated at 0, 24, and 48 h. Two experimental conditions were applied 1) unadapted condition in which *S*. Typhimurium was added at the beginning of the culture incubation and 2) adapted condition in which *S*. Typhimurium was added after a 24 hour pre-incubation of the cecal bacteria with the feed and/or rice bran. Among the three rice brans, only Calrose exhibited a rapid inhibition of *S*. Typhimurium, which decreased to undetectable levels after 24 h under the adapted incubation. Changes in microbiological composition and metabolites by addition of Calrose bran were also investigated with an Illumina MiSeq platform and gas chromatography—mass spectrometry, respectively. Addition of Calrose bran resulted in significant changes including decreased Firmicutes phylum abundance and an increased number of metabolites associated with fatty acid metabolism. In summary, it appears that rice bran from specific rice cultivars may be effective as a means to reduce *Salmonella* in the chicken ceca. In addition, Calrose rice bran inclusion leads to changes in cecal microbiological composition and metabolite profile.

## Introduction

Ensuring the microbiological safety of poultry products is a critical concern for the poultry industry. Traditional prebiotics, were defined as “a non-digestible ingredient which beneficially influence the host by selectively stimulating the growth and/or activity of one or a limited number of bacteria in the colon”, and promoted occasionally as effective alternatives to antimicrobials. More recently, the definition of a prebiotic has been modified and now includes a broader range of ingredients derived from multiple sources [1, 2]. Given the escalating global public health concern of antibiotic resistance (in *Salmonella*), prebiotics and their resultant modulation of gastrointestinal microbiota activity can thus be promoted as potential alternatives to antimicrobial therapy use in poultry [3–6]. Rice bran is an underutilized product of rice milling and contains a variety of components including proteins, amino acids, complex carbohydrates, minerals, vitamins, phytonutrients, phospholipids, essential fatty acids, and antioxidants that have nutritional value [7]. Rice bran also possesses various components that exhibit prebiotic activities which can modulate microbiota in the intestine and potentially help to prevent chronic diseases [8], and evidence supports that the antimicrobial activity of rice bran against *Salmonella* Typhimurium can vary across cultivars [9]. Rice bran has been investigated for its prebiotic properties and several studies have demonstrated that *S*. Typhimurium colonization in the animal gastrointestinal tract can be reduced by inclusion of rice bran in the diet [10–12]. There is no information on the general ability of rice bran to reduce *Salmonella* when fed to poultry, or on the relative potency of brans derived from different rice cultivars in specifically reducing *Salmonella* in the poultry gastrointestinal tract.

The main site of *Salmonella* colonization in poultry is the ceca [13]. Poultry ceca contain a variety of bacteria which are characterized as strict anaerobes [14–16]. To investigate the possible effects of prebiotics or prebiotic-like materials on these anaerobic bacteria and their interaction with prebiotics and *Salmonella*, an anaerobic mixed culture system has been employed as a screening tool [17].

Inhibition of *S*. Typhimurium by rice bran was previously investigated across rice varieties in mice [9, 18], and these findings are relevant to the present study for chickens as the previous studies compared three different cultivars of rice: Jasmine, Red Wells, and Calrose that were examined as treatment candidates in the current study. The previous studies confirmed variation in secondary metabolite components such as total phenolics, γ-oryzanol, fatty acids and vitamin E isoforms [9, 18, 19]. Jasmine rice has a brown bran layer and has significantly less total phenolic content compared to Red Wells, but is relatively rich compared to other varieties in γ-tocotrienol, a vitamin E isoform with anti-proliferative activity on Caco-2 cells, a human colon cancer cell line [19]. Red Wells is isogenic with the agronomically important Wells variety, except for a deletion mutation that restores the correct reading frame in the *Rd* gene [20], encoding a regulatory factor that activates synthesis of proanthocyanidins, precursors of anthocyanins, flavonoids, and flavonoid derivatives [21]. The wild-type Rd restores the red pericarp color of wild type red rice, thus the name Red Wells. Calrose rice contains a brown bran and this bran has been shown to exhibit a synergistic effect with probiotics on the elimination of rotavirus and norovirus-induced diarrhea in orally-challenged gnotobiotic neonatal pigs [22, 23].

In this study, we used cecal contents collected from broiler chickens, which possess a fairly diverse microbiota, to investigate empirical effects of different rice bran on reducing the *Salmonella* population. In addition, because the microbiota in ceca can change as the host matures [1], cecal materials were obtained and analyzed for microbiome changes at two different chicken ages (28 and 42 days). Metabolomic analysis by gas chromatography- mass spectrometry (GC-MS) of 24 h anaerobic cultures with and without rice bran was also investigated to examine metabolites of rice bran or of rice bran fermentation that might have roles in reducing *Salmonella* growth. Together with Illumina MiSeq DNA sequencing of 16s ribosomal DNA from these cultures, we present a comprehensive view of rice bran effects in a controlled system that approximates the anaerobic conditions and microbiota of the chicken hindgut. The findings indicate the potential utility of rice bran or components thereof in the control of *Salmonella* in the preharvest broiler chicken, and contribute to a better understanding of ecological and metabolic changes in the ceca upon exposure to rice bran.

## Materials and methods

### Bacterial strain

*Salmonella enterica* serovar Typhimurium marker strain ST97, a nalidixic acid-resistant (NA^R^) strain, was kindly provided by Dr. Billy Hargis, Department of Poultry Science, University of Arkansas (Fayetteville, AR). The bacterial strain was grown in sterile tubes containing Luria-Bertani (LB) medium supplemented with 20 μg/ml nalidixic acid at 37°C for 16 h with agitation at 250 rpm.

### Rice bran cultivars

Heat-stabilized rice bran of cultivars Jasmine, Red Wells, and Calrose were obtained from Dr. Elizabeth Ryan, Colorado State University. Rice bran isolation was performed following detailed described in previously published research [19].

### Anaerobic culture medium

The chicken cecal microbiota were inoculated from freshly harvested chicken ceca at a 1:3000 dilution into anaerobic dilution solution (ADS:,0.45g/L K_2_HPO_4_, 0.45 g/L KH_2_PO_4_, 0.45 g/L (NH_4_)_2_SO_4_, 0.9 g/L NaCl, 0.1875 g/L MgSO_4_-7H_2_O, 0.12 g/L CaCl_2_-2H_2_O, 1 ml/L 0.1% resazurin, 0.05% cysteine-HCl, and 0.4% sodium carbonate) [16, 24–28] with ground (25 mesh) Torres chick starter feed (1.25% w/v) as the carbon source. Prepared ADS was sparged with an anaerobic gas mixture consisting of 90% nitrogen/5% carbon dioxide/5% hydrogen in an anaerobic chamber for 30 minutes using an aquarium air pump and airstone before autoclaving. After autoclaving, the ADS was subsequently cooled to room temperature and placed in an anaerobic chamber overnight to remove all traces of oxygen. One bird was used as cecal donor in each of three independent experiments.

### Cecal inocula preparation

Cecal contents were obtained from freshly killed 28-day and 42 day-old Cobb male broiler chickens (Cobb-Vantress, Siloam Springs, AR), killed by CO_2_ asphyxiation, followed by aseptically removing the ceca using sterile tools, and the ceca were subsequently placed in sterile sample bags in a portable anaerobic box (Mitsubishi Gas Chemical Co., Japan) containing oxygen-scrubbing sachets. The use of chickens as a source for cecal inocula was approved by a University of Arkansas Institutional Animal Care and Use Committee (IACUC) to ensure humane treatment of the chickens. Immediately after harvest, ceca were transferred to an anaerobic chamber (Coy Laboratory Products, Grass Lake, MI) devoid of oxygen and containing an atmosphere of 90% nitrogen/5% CO_2_/5% hydrogen. To maintain an anaerobic environment inside the chamber, two palladium catalyst scrubbers were maintained continuously.

### Anaerobic *in vitro* mixed cultures

**Fig 1:**
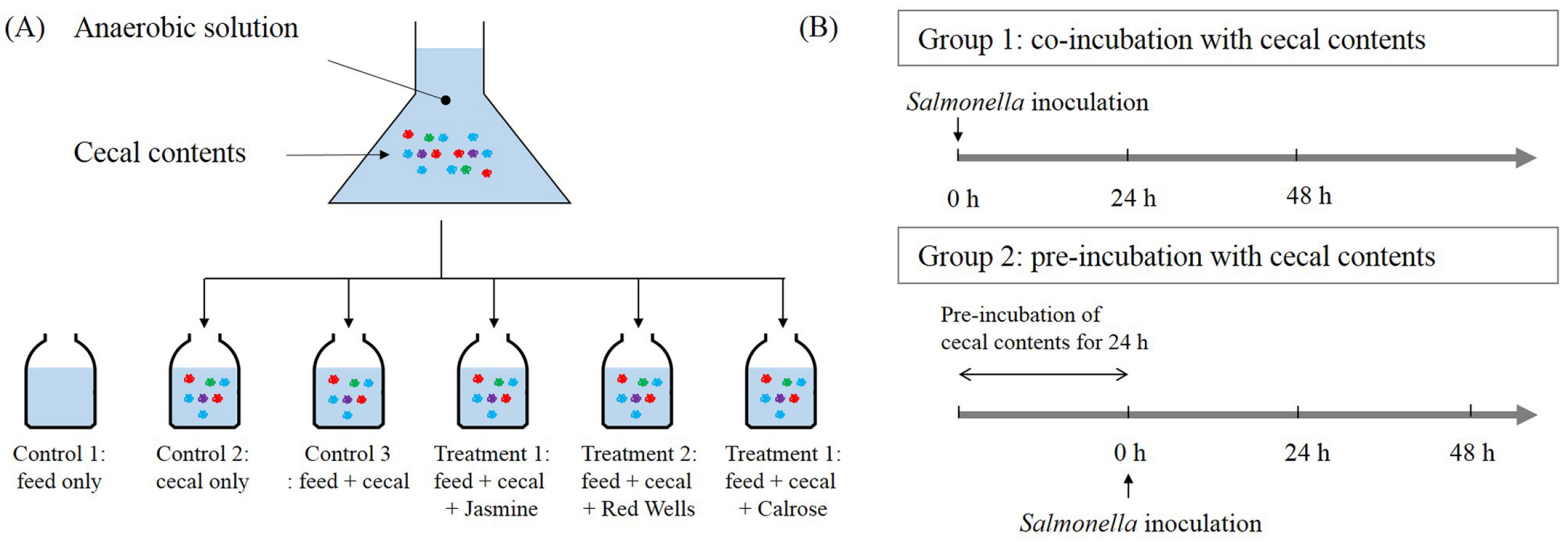
Experimental design of the anaerobic *in vitro* mixed cultures. (A) Controls contained 1) feed, 2) cecal content, and 3) feed and cecal contents without rice bran while treatments contained feed, cecal content, and different amounts of rice bran including Jasmine, Red Wells, and Calrose. (B) Experimental design used in this study. Unadapted condition: cultures receive *Salmonella* Typhimurium at the same time as cecal content (at 0 time). Adapted condition: cultures received *Salmonella* Typhimurium after a 24 h incubation of cecal content under anaerobic conditions. This figure was adapted and modified from Rubinelli et al. (2016) [17]

The overall experimental approach used in this study is shown in Fig 1. Briefly, a portion of the cecal contents were weighed within the aseptic chamber. The weights of 0.1 gram aliquots of cecal contents were measured and subsequently diluted 1:3000 by addition to 300 ml ADS. Prior to the experiment, the absence of NA resistant *S*. Typhimurium was confirmed by inoculation into tetrathionate (TT) enrichment broth (BD Biosciences) followed by streaking onto brilliant green agar plates (BD Biosciences). A 20 ml portion of the diluted cecal content was added to each sterile serum bottle and 0.25 g chicken feed and/or 0.2 g rice bran was added to the bottles as indicated in Fig 1A. Three control groups were used in this study: ADS containing 1) feed only, 2) cecal content only, and 3) feed and cecal content combined. Three types of rice bran cultivars (Jasmine, Red Wells, and Calrose) were added to the anaerobic mixed cultures (1% w/v) as treatment groups.

### *Salmonella* inoculation and enumeration of survivors

The cell density of a 16 h culture of NA-resistant *S*. Typhimurium (strain ST97) was measured by spectrophotometry at 600 nm. Working in the anaerobic chamber, this culture was then added to each 20 mls of diluted cecal content in ADS to yield approximately 1 x 10^7^ CFU/ml starting density of *S*. Typhimurium. A small portion of the culture was removed for plating, and the cultures were then stoppered with airtight rubber stoppers and aluminum crimps, removed from the anaerobic chamber, and incubated at 37°C for 48 h in a shaking incubator (Model G25, New Brunswick, Inc.) at 150 rpm. Two different experimental designs were established as shown in Fig 1B, referred to as Group 1 and Group 2. In Group 1 (unadapted incubation), *S*. Typhimurium was added at the beginning of the culture incubation along with cecal bacteria and/or chicken feed and/or rice bran. In Group 2 (adapted incubation), *S*. Typhimurium was added after a 24 h pre-incubation of the cecal bacteria with the chicken feed and/or rice bran. After 0, 24, and 48 h incubation, an aliquot of each culture was removed and subsequently diluted with sterile phosphate-buffered saline (PBS). Undiluted and diluted aliquots of cultures were spread in duplicate on Brilliant Green agar plates (BD Biosciences) supplemented with 20 μg/ml NA for quantification of colony forming units (CFU) of *S*. Typhimurium. If no *S*. Typhimurium were detected at a particular time point in the undiluted culture, this culture was inoculated into tetrathionate (TT) enrichment broth in order to confirm that no *S*. Typhimurium survived. Experiments were performed in triplicate.

### Microbiome analysis

Microbiome analysis was conducted in triplicate on two controls (“feed + cecal” and “cecal only” controls) and the Calrose treatment (feed + cecal + Calrose) in the adapted mixed culture condition described above at various time points (0, 6, 12, 24, and 48 h) using cecal contents from two different ages of broilers (28 and 42 days). Bacterial genomic DNA was extracted via a QIAamp Fast DNA Stool Mini Kit (Qiagen, Valencia, CA) following the manufacturer’s instructions. Sequencing was performed by targeting the V4 hypervariable region of 16S rRNA using an Illumina MiSeq platform (Illumina, San Diego, CA) and sequence reads were analyzed with the Quantitative Insights into Microbial Ecology (QIIME) pipeline (version 1.9.0) as described previously [29].

### Metabolite analyses

#### Extraction of metabolites

Three replicate anaerobic cultures containing 1% Calrose rice bran, 1.25% chicken feed, and a 1:3000 dilution of chicken cecal contents in ADS were grown for 24 h at 37°C in airtight serum bottles with thick stoppers and crimps and contained a 5% H_2_/5% CO_2_/90% N_2_ atmosphere. Control cultures were identical except that they did not contain rice bran. The cecal contents used were that of three 6-week-old broiler chickens (three biological replicates). After 24 h, each culture was centrifuged at 8000 x g for 6 minutes. The remainder of the metabolomics analysis was performed at the University of California Davis Genome Center, Metabolomics Core and Research Laboratories, as follows: A 30 μl portion of each supernatant was extracted with 1 ml of Extraction Solution (acetonitrile: isopropanol: water, 3:3:2, de-gassed with nitrogen for 5 minutes and pre-cooled to -20°C). This mixture was subsequently vortexed for 10 seconds and shaken for 5 minutes at 4°C using an orbital mixer. The resulting mixture was centrifuged 2 min, 14,000 x g. A 500 μl portion of supernatant was dried using a centrivap (Labconco). The dried sample was derivatized and analyzed by GC-MS as described in the following subsections.

#### Injector conditions

The Agilent 6890 GC was equipped with a Gerstel automatic liner exchange system (ALEX) that includes a multipurpose sample (MPS2) dual rail, and a Gerstel CIS cold injection system (Gerstel, Muehlheim, Germany) with a temperature program as follows: 50°C to 275°C final temperature at a rate of 12°C/s and held for 3 minutes. Injection volume was 0.5 μl with 10 μl/s injection speed on a splitless injector with purge time of 25 seconds. Liner (Gerstel #011711-010-00) was changed after every 10 samples, (using the Maestro1 Gerstel software vs. 1.1.4.18). Before and after each injection, the 10 μl injection syringe was washed three times with 10 μl ethyl acetate.

#### Gas chromatography conditions

A 30 m long, 0.25 mm i.d. Rtx-5Sil MS column (0.25 μm 95% dimethyl 5% diphenyl polysiloxane film) with an additional 10 m integrated guard column was used (Restek, Bellefonte PA). 99.9999% pure Helium with built-in purifier (Airgas, Radnor PA) was set at a constant flow of 1 ml/min. The oven temperature was held constant at 50°C for 1 min and then ramped at 20°C/min to 330°C and finally held constant for 5 min.

#### Mass spectrometry

A Leco Pegasus IV time of flight mass spectrometer was controlled by the Leco ChromaTOF software vs. 2.32 (St. Joseph, MI). The transfer line temperature between gas chromatograph and mass spectrometer was set to 280°C. Electron impact ionization at -70 eV was employed with an ion source temperature of 250°C. Acquisition rate was 17 spectra/second, with a scan mass range of 85 to 500 Da. The column was a Restek corporation Rtx-5Sil MS (30m x 0.25 mm I.D. with 0.25 μm 95% dimethyl/5% diphenylpolysiloxane film). The mobile phase was helium, column temperature 50 to 330°C, flow rate 1 ml/min. Injection volume was 0.5 ml, injection temperature 50°C ramped to 250°C at 12°C/s. Oven temperature program was 50°C 1 min, then ramped at 20°C/min to 330°C, and finally held for 5 min.

#### Detection and identification of metabolites and metabolic pathway classification

Raw data files were preprocessed directly after data acquisition and stored as ChromaTOF-specific *.peg files, as generic *.txt result files and additionally as generic ANDI MS *.cdf files. ChromaTOF vs. 2.32 was used for data preprocessing without smoothing, 3 s peak width, baseline subtraction just above the noise level, and automatic mass spectral deconvolution and peak detection at signal/noise levels of 5:1 throughout the chromatogram. Apex masses were reported for use in the BinBase algorithm. Result *.txt files were exported to a data server with absolute spectra intensities and further processed by a filtering algorithm implemented in the metabolomics BinBase database. The BinBase algorithm (rtx5) used the settings: validity of chromatogram (< 10 peaks with intensity >10^7^ counts s^-1^), unbiased retention index marker detection (MS similarity > 800, validity of intensity range for high m/z marker ions), retention index calculation by 5th order polynomial regression. Spectra were cut to 5% base peak abundance and matched to database entries from most to least abundant spectra using the following matching filters: retention index window ± 2,000 units (equivalent to approximately ± 2 s retention time), validation of unique ions and apex masses (unique ion must be included in apexing masses and present at greater than 3% of base peak abundance), mass spectrum similarity must fit criteria dependent on peak purity and signal/noise ratios and a final isomer filter. Metabolites were classified into possible pathways using the KEGG and PubChem databases.

### Statistical analysis

The log CFU/ml for control (feed only, cecal only, feed + cecal) and experimental treatments (feed + cecal + Jasmine, Red Wells, or Calrose rice bran) were determined by averaging all biological replicates and one way analysis of variance (ANOVA) was conducted to compare differences of bacterial population or relative abundance among groups with JMP Genomics 7.0 (SAS Institute Inc., Cary, NC) at *P* < 0.05. A Student’s t-test was also used to compare mean abundances of metabolites between Calrose and the “no rice bran” control (NC). *P* < 0.05 in a two-tailed test was considered significant.

## Results and discussion

### Calrose decreased *Salmonella* Typhimurium survival in anaerobic mixed culture during adapted incubation conditions

This study investigated effect of rice brans within two different conditions including Group 1 (unadapted) and Group 2 (adapted condition). The survival of *S*. Typhimurium in the anaerobic mixed culture containing different rice brans (Jasmine, Red Wells, and Calrose) under unadapted and adapted incubation conditions is shown in Table 1. In the unadapted condition, neither of the controls or the experimental treatments significantly inhibited *S*. Typhimurium after either 24 or 48 h incubation. The population of *S*. Typhimurium was increased during incubation from 6.73 to 6.97 log CFU/ml (initial population) to 7.26 to 8.78 log CFU/ml after 24 h and 7.27 to 8.60 log CFU/ml after 48 h.

**Table 1:** Population of *Salmonella* Typhimurium (log CFU/ml) in an anaerobic *in vitro* mixed culture.

Experimental condition	Treatment	Incubation time (hours)		
		0	24	48
Unadapted condition	Control 1: feed only	6.97 ± 0.09	8.25 ± 0.09^b^	8.02 ± 0.26^a^^b^
	Control 2: cecal only	6.78 ± 0.15	7.26 ± 0.12^c^	7.27 ± 0.07^b^
	Control 3: feed + cecal	6.88 ± 0.07	8.49 ± 0.11^a^^b^	8.51 ± 0.09^a^
	Treatment 1: feed + cecal + Jasmine	6.73 ± 0.10	8.70 ± 0.09^a^	8.60 ± 0.13^a^
	Treatment 2: feed + cecal + Red Wells	6.86 ± 0.07	8.61 ± 0.10^a^	8.55 ± 0.09^a^
	Treatment 3: feed + cecal + Calrose	6.92 ± 0.12	8.78 ± 0.08^a^	7.76 ± 0.64^a^^b^
Adapted condition	Control 1: feed only	6.84 ± 0.14	7.19 ± 0.04^a^	6.95 ± 0.13^a^
	Control 2: cecal only	6.65 ± 0.12	6.35 ± 0.30^a^	5.78 ± 0.91^a^
	Control 3: feed + cecal	6.78 ± 0.13	6.20 ± 0.08^a^	3.91 ± 0.72^b^
	Treatment 1: feed + cecal + Jasmine	6.75 ± 0.14	5.17 ± 1.24^a^	5.78 ± 0.39^a^^b^
	Treatment 2: feed + cecal + Red Wells	6.65 ± 0.07	5.11 ± 1.20^a^	5.74 ± 0.28^a^^b^
	Treatment 3: feed + cecal + Calrose	6.73 ± 0.07	2.55 ± 0.82^b^	ND^c^^,^*

* ND, not detected (detection limit: 10 CFU/ml).

^a–c^Mean values in the same column and experimental condition denoted by different superscript letters represent statistically significant differences (*P* < 0.05).

Average log CFU data comes from 3 independent experiments using different cecal contents.

In contrast, *S*. Typhimurium survival was significantly reduced under the adapted incubation conditions (Group 2 treatments) by Calrose rice bran compared to the control with feed + cecal contents but no rice bran (Table 1). Calrose bran rapidly decreased *S*. Typhimurium levels when compared with the feed + cecal control at 24 h. The population of *S*. Typhimurium (initially 6.73 log CFU/ml) in the Calrose bran-containing cultures were significantly reduced to 2.55 log CFU/ml after 24 h (*P* < 0.05). However, the level in the feed + cecal control at this same timepoint was 6.20 log CFU/ml. The Calrose bran-containing cultures reached an undetectable level (below the limit of detection of the dilution plating less than 10 CFU/ml) after 48 h, while the feed + cecal control cultures and Jasmine- and Red Wells-containing cultures were not significantly different from each other and remained much higher (approx. 4 to 6 log CFU/ml, Table 1).

Cultures after 48 h in the presence of Calrose bran yielding no detectable colonies were inoculated into TT enrichment broth to determine if *S*. Typhimurium could be recovered from them and detected by subsequent streak plating from the enrichments. However, none of these cultures exhibited detectable growth after inoculation from TT enrichment broth, suggesting that *S*. Typhimurium was completely eradicated by adding Calrose to the anaerobic mixed culture.

Calrose rice bran-treated anaerobic mixed cultures consistently and reproducibly exhibited a rapid killing of *S*. Typhimurium, but only under adapted cecal microbiota conditions. Different results between unadapted and adapted conditions suggest that the fermentation associated with the cecal 24 h pre-incubation of cecal contents prior to addition of *S*. Typhimurium may play an important role in inhibiting *S*. Typhimurium in the anaerobic mixed culture. Collectively, these results suggest that incubation of the cecal microbiome in the presence of Calrose rice bran prior to inoculation of pathogen influences microbiota and/or fermentation profiles that work prophylactically to prevent *Salmonella* colonization after infections [9, 12]. These results, which demonstrate that higher levels of *Salmonella* growth inhibition by Calrose are achieved in adapted conditions, are also in accordance with our previous study reporting that a commercial biological product derived from yeast fermentation showed significantly higher bactericidal effects in the adapted condition compared to the unadapted condition [17]. This supports that under the adapted conditions, the yeast product and Calrose might have similar downstream effects on *Salmonella* growth.

### Microbiome

#### Microbial correlation among groups

**Fig 2:**
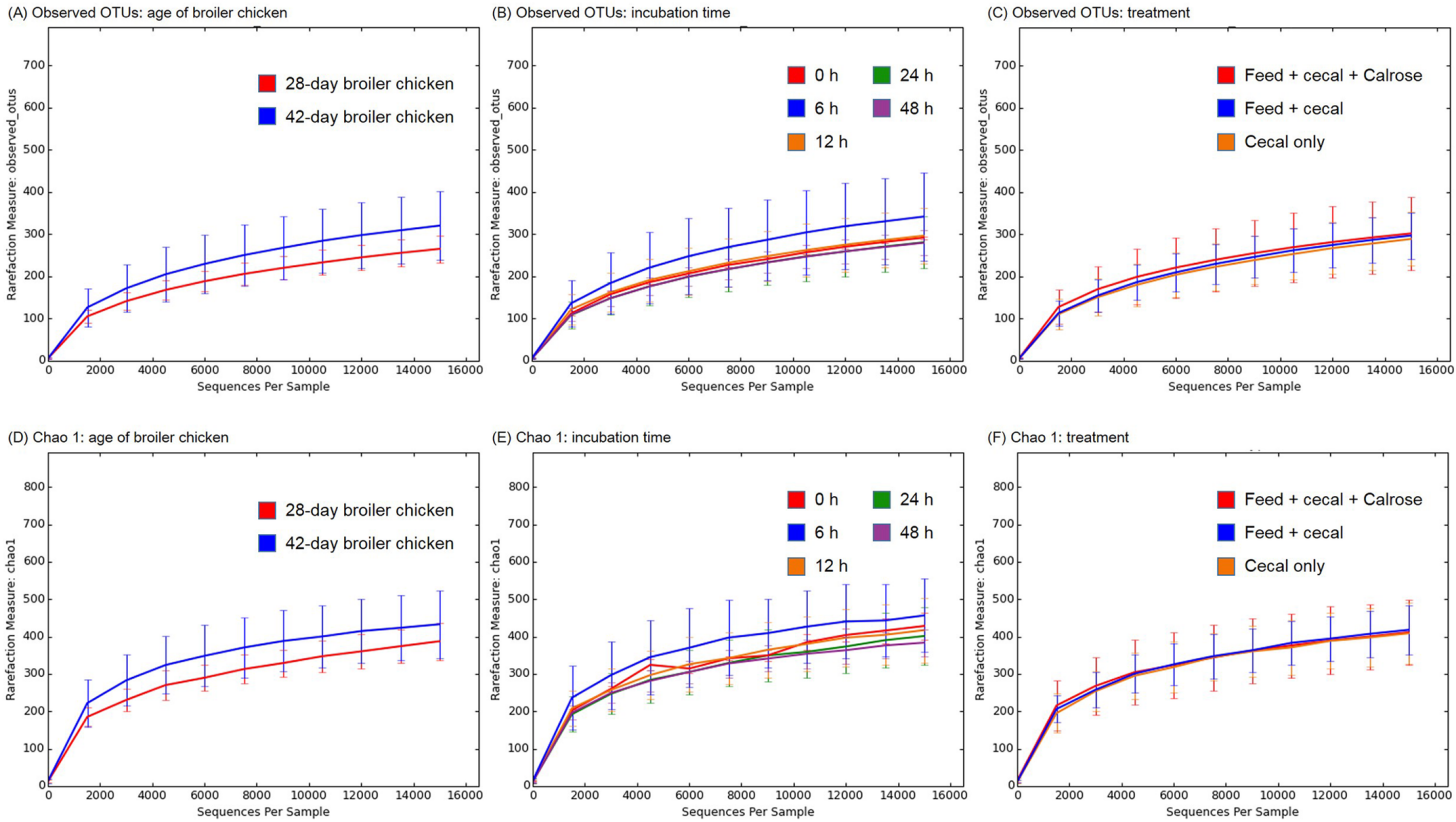
Alpha diversity analysis among groups. Rarefaction curves of (A-C) Observed_OTUs and (D-F) Chao 1.

Rarefaction plots of average observed OTUs and Chao1 from alpha diversity analysis are shown in Fig 2. Samples containing cecal contents from 42 day old broiler chickens exhibited greater observed OTU numbers compared to the other samples containing cecal contents from 28 day old broiler chickens (Fig 2A). Samples after 6 h incubation exhibited greater numbers than the other samples (Fig 2B). There were no significant differences in OTU numbers among cecal only control, feed + cecal control, and Calrose treatment group (Fig 2C). The Chao 1 rarefaction plot estimating species richness revealed similar results with the observed_OTU plot (Fig 2D–2F).

**Fig 3:**
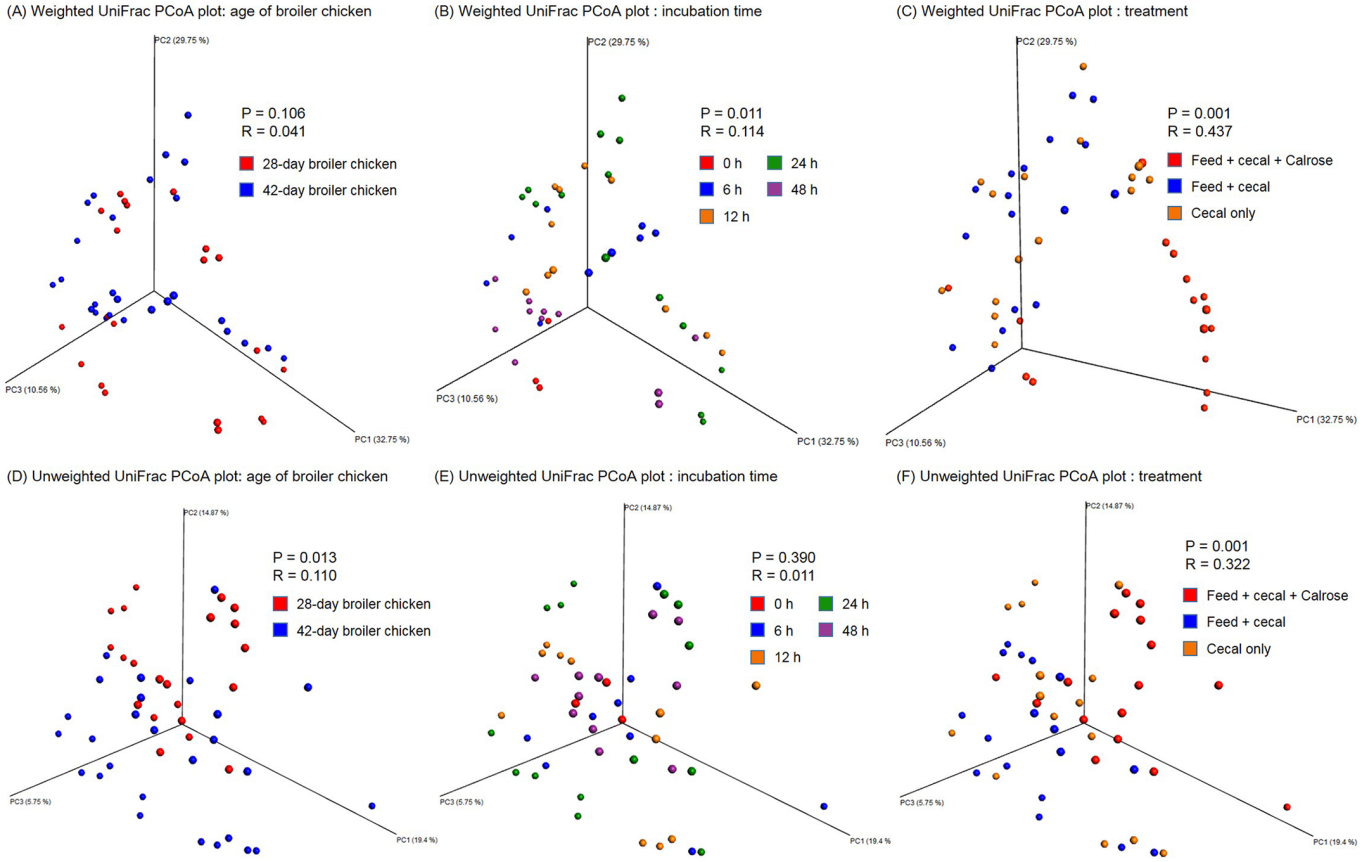
Beta diversity analysis among groups. (A-C) Weighted and (D-F) unweighted UniFrac PCoA plots of individual sample in each group.

The Fig 3 represents weighted (A-C) and unweighted (D-F) principal coordinated analysis (PCoA) UniFrac plots generated by the beta diversity analysis. In the PCoA UniFrac plot, each data point represents an individual sample. The P value indicates groups that are significantly different and the R value indicates how strongly groups are different from each other. An R value near 0 meant no separation while the R value close to 1 was used to indicate that there was dissimilarity among groups. An R value from both weighted and unweighted PCoA plots categorized by age of the broiler chicken and incubation time (Fig 3A, 3B, 3D and 3E) was near 0 (0.011 to 0.114) which implied no significant dissimilarity among groups by these parameters. In both plots categorized by treatment (cecal only control, cecal + feed control, and Calrose treatment), only the Calrose treatment group was slightly clustered but detectable patterns of obvious clustering were not observed (R value from weighted and unweighted plot: 0.437 and 0.322, respectively) (Fig 3C and 3F).

#### Comparison of bacterial communities

**Fig 4:**
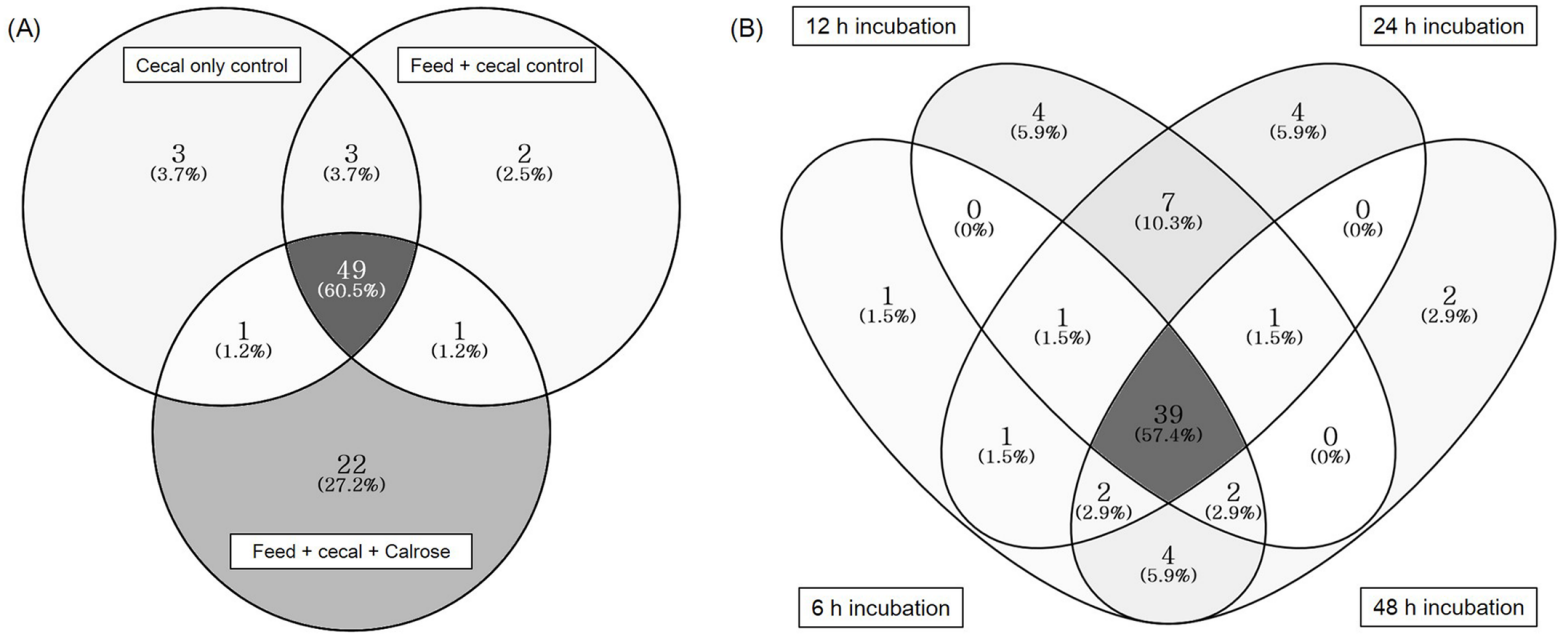
Distribution of bacterial taxa in genus level in sample groups. (A: controls and treatment, B: incubation time).

The overlap in microbial taxa at the genus level in control groups and Calrose treatment group are shown in Fig 4A as Venn diagrams. A 60.5% proportion of the bacterial genera were present in both control groups and the Calrose treatment group; indicating that controls and treatment groups shared similar bacterial communities. Only 3 and 2 genera were unique in cecal only control and feed + cecal control, respectively. In contrast, 22 unique genera were exclusively present in the Calrose treatment group but not in the control groups. This highlights the fact that Calrose treatment can significantly alter microbial communities. Venn diagram by incubation time is also shown in Fig 4B. More than half of the genera (57.4%) were common among the four groups (6, 12, 24, and 48 h incubation groups).

#### Rice bran treatments and adaptation conditions are associated with phyla shifts in the microbiome profiles of incubated cecal contents originating from 28-day broiler chickens

**Fig 5:**
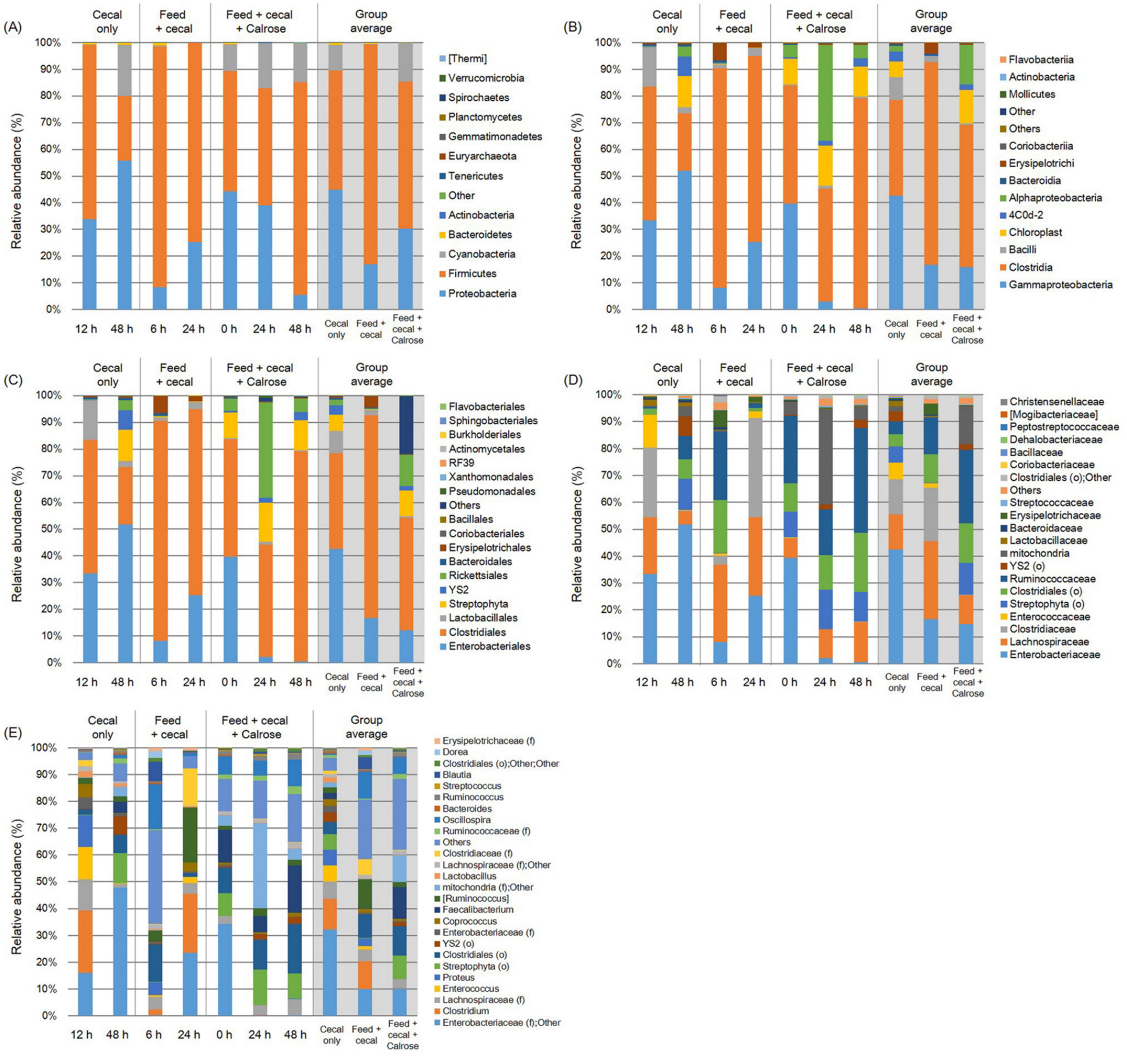
Relative abundance of major bacteria among different treatment groups at (A) phylum, (B) class, (C) order, (D) family, and (E) genus level in anaerobic mixed cultures (adapted condition) containing cecal contents. Microbiome analysis was conducted in triplicate on cecal contents only, feed and cecal contents, and feed + cecal + Calrose at various time points using cecal contents from 28 day-old broiler chickens. The bars represent the standard deviation. “f” and “o” in parentheses indicate family and order, respectively.

Microbiome analysis provides comprehensive information on bacterial composition changes in cecal microbiota when exposed to dietary changes or other factors that may influence microorganisms in the gastrointestinal tract. The relative abundance of major bacterial groups from phylum to genus level in an anaerobic mixed culture batch system containing cecal contents obtained from 28 days of broiler chickens is shown in Fig 5. Some data groups (e.g. cecal only control after 0, 6, and 24 h and feed + cecal control after 0, 12, 48 h) were removed since they yielded sequencing read numbers that were too low during sequencing and QIIME analysis.

The overall microbiota of each group revealed generally similar patterns; the top three dominant bacterial groups at the phylum level belonged to either Firmicutes, Proteobacteria, or Cyanobacteria (Fig 5A). The cecal only control group was dominated by Proteobacteria (group average value, 44.80%), Firmicutes (44.78%), and Cyanobacteria (9.62%) accounting for 99.20% of the entire phyla. However, the most predominant bacterial group in the feed + cecal control and Calrose treatment was Firmicutes with 82.37 and 42.93%, respectively. In the Calrose treatment group, the relative abundance of Firmicutes was significantly increased (P < 0.05) with increased incubation time from 44.88% (initial) and 43.90% (24 h) to 79.60% (48 h).

Sequenced microbiota recovered from each group exhibited different class levels (Fig 5B). In the cecal only control, Clostridia (group average value, 35.80%), Gammaproteobacteria (42.71%), and Bacilli (8.48%) were relatively common accounting for 87.00%. In contrast, the Calrose treatment group harbored the greatest proportion of Clostridia constituting 42.14% and also contained Gammaproteobacteria and Alphaproteobacteria at 12.56 and 11.67%, respectively. In the Calrose treatment group, when the incubation time increased, the abundance of Clostridia significantly increased from 44.09% at initial time to 78.70% at 48 h incubation while that of Gammaproteobacteria decreased from 39.77% at the initial time to 0.58% at 48 h incubation.

At the order taxonomic level, overall microbial distributions via sequencing exhibited the most operational taxonomic units (OTUs) belonging to Clostridiales and Enterobacteriales with 35.80 and 42.68% for the cecal only control group, 75.93 and 16.71% for the feed + cecal control, and 42.14 and 12.31% for the Calrose treatment group (Fig 5C). The Calrose treatment group revealed higher levels of Rickettsiales (11.61%) than the other control groups (2.07 and 0.13% for cecal only and feed + cecal control, respectively). Along with incubation time, the proportion of Clostridiales in the Calrose treatment group generally increased (from 44.09 at 0 h to 78.70% at 48 h) while Enterobacteriales significantly decreased (from 39.69 at 0 h to 0.49% at 48 h).

Relative abundance of major bacterial groups at the family taxonomic level varied among different treatments. The top 5 bacterial groups of the cecal only control were *Enterobacteriaceae* (group average value, 42.68%), *Clostridiaceae* (13.07%), *Lachnospiraceae* (13.10%), *Enterococcaceae* (6.21%), and Streptophyta (order level) (5.97%). *Lachnospiraceae* (29.54%), *Clostridiaceae* (20.24%), *Enterobacteriaceae* (16.73%), *Ruminococcaceae* (13.89%), and Clostridiales (order level) (10.78%) were common in feed + cecal control groups. For the Calrose treatment groups, *Ruminococcaceae*, Clostridiales (order level), *Enterobacteriaceae*, Streptophyta (order level), and *Lachnospiraceae* accounted for the highest abundance level with 22.89, 12.40, 12.31, 9.82, and 8.99%, respectively. During prolonged incubation, the proportion of *Lachnospiraceae*, *Ruminococcaceae*, and Clostridiales (order level) increased (7.31 to 15.20%, 25.14 to 39.55%, and 10.76 to 22.23%, respectively) while that of *Enterobacteriaceae* decreased (39.63 to 0.49%).

Similar to the other taxonomic hierarchy, different treatments also exhibited variable proportions in the primary detectable bacterial genera. The top 5 genera were *Enterobacteriaceae* (family level); Other, *Clostridium*, *Lachnospiraceae* (family level), *Enterococcus*, and *Proteus* for the cecal control group and *Ruminococcus*, *Clostridium*, *Oscillospira*, *Enterobacteriaceae* (family level); Other, Clostridiales (order level) for feed + cecal control group. In contrast, *Faecalibacterium* (13.51%), Clostridiales (order level) (12.40%), *Enterobacteriaceae* (family level); Other (11.58%), Streptophyta (order level) (9.82%), and *Oscillospira* (7.18%) were apparently present in the Calrose treatment groups as the primary genera. There were significant increases in abundance level of Clostridiales (order level) and *Faecalibacterium* from 10.76 to 22.23% and 13.85 to 21.35%, respectively, along with incubation time.

#### Microbiome profiles of incubated cecal contents originating from 42-day broiler chickens

**Fig 6:**
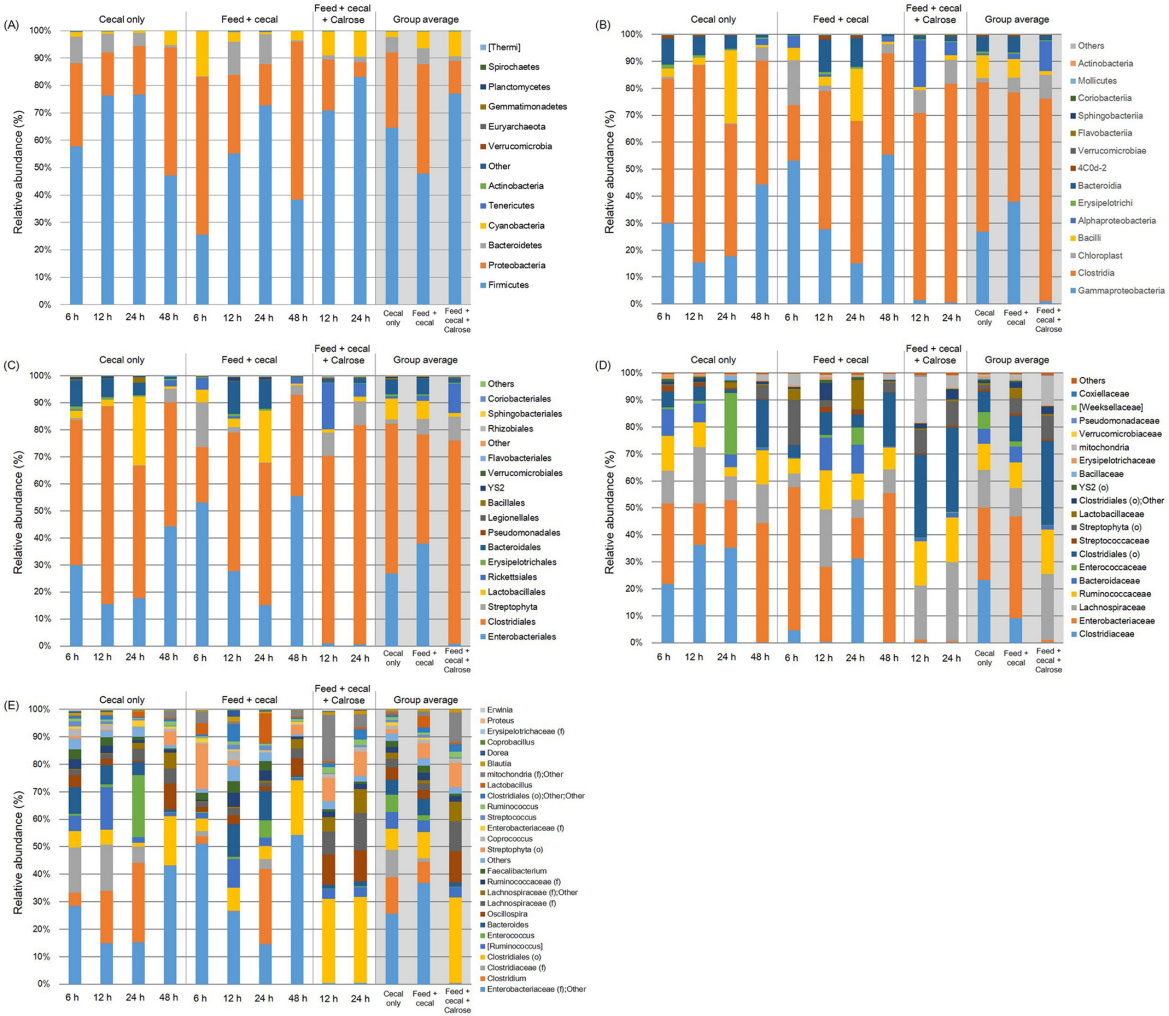
Relative abundance of major bacteria among different treatment groups at (A) phylum, (B) class, (C) order, (D) family, and (E) genus level in anaerobic mixed cultures (adapted condition) containing cecal contents. Microbiome analysis was conducted in triplicate on cecal contents only, feed and cecal contents, and feed + cecal + Calrose at various time points using cecal contents from 48 day-old broiler chickens. The bars represent the standard deviation. “f” and “o” in parentheses indicate family and order, respectively.

The relative abundance of major bacteria in different groups from phylum to genus level in an anaerobic mixed culture containing cecal contents obtained from 42 day old broiler chickens is shown in Fig 6. Firmicutes, Proteobacteria, Bacteroidetes, and Cyanobacteria were the major bacterial groups of the identified phyla (Fig 6A). Relative abundance of Proteobacteria in the Calrose treatment (group average value, 12.06%) was generally lower than that of feed + cecal control (39.74%) while abundance of Firmicutes was higher than feed + cecal control (79.93 versus 47.94%). In the Calrose treatment, the abundance of Firmicutes was increased (70.80% at 12 h to 83.06% at 24 h). The relative abundance of Proteobacteria in Calrose treatment was 18.71 after 12 h incubation and 5.41% after 24 h incubation, respectively. These changes (an increase in Firmicutes and decrease in Proteobacteria) are in accordance with the results from Calrose treatments using 28 days cecal contents.

At the class taxonomic level, the top two dominant bacterial groups for the cecal only and feed + cecal control treatments were Clostridia and Gammaproteobacteria with 55.41 and 26.86% (group average value) for cecal only and 40.46 and 37.89% for feed + cecal control, respectively (Fig 6B). In the Calrose treatment group, the most predominant bacterial group was Clostridia accounting for 75.19% but the relative abundance of Gammaproteobacteria was significantly lower (1.01%) than the control groups. Bacilli were also relatively common in control groups (8.41 and 6.99%, respectively) while their abundance was significantly less in the Calrose treatment (1.45%). In contrast, the proportion of Alphaproteobacteria was higher in the Calrose treatment (11.02%) than the control groups (0.70 and 1.85%). During incubation, the proportion of Clostridia was maintained at a high level (69.32 and 81.05% at 12 and 24 h).

Sequenced microbiota recovered from each treatment exhibited proportionally different order taxonomic levels as well (Fig 6C). The top two dominant bacterial groups at the order level belonged to either Clostridiales or Enterobacteriales. The cecal only control and feed + cecal control treatments were dominated by Clostridiales (group average value, 55.41 and 4046%, respectively), Enterobacteriales (26.84 and 37.83%), and Lactobacillales (7.82 and 6.87%). However, the most predominant bacterial groups occurring in the Calrose treatment mixed cultures were Clostridiales (75.19%) and Rickettsiales (10.92%). The proportion of Clostridiales was significantly higher for the Calrose treatment than controls while the abundance of Enterobacteriales was significantly lower in the Calrose treatment (0.97%).

At the family taxonomic level, overall microbial distributions detected by sequencing revealed that most of the identified OTUs belonged to *Clostridiaceae*, *Enterobacteriaceae*, *Lachnospiraceae*, and *Ruminococcaceae* with 23.25, 26.84, 14.07, and 9.55% (group average value) for the cecal only control group and 9.03, 37.83, 10.50, and 9.45% for feed + cecal control, respectively (Fig 6D). In the Calrose treatment group, mixed culture incubations yielded a relatively high percentage of *Clostridiales* (39.19%), *Lachnospiraceae* (24.62%), and *Ruminococcaceae* (16.43%). The proportion of Enterobacteriaceae was significantly lower for the Calrose treatment (0.12%) than control groups (23.25% for cecal only control and 9.03% for feed + cecal control).

The different treatments also exhibited variable proportions in the primary bacterial genera. The top 5 genera were *Enterobacteriaceae* (family level); Other, *Clostridium*, *Clostridiaceae* (family level), Clostridiales (order level), and *Ruminococcus* for the cecal control group *Enterobacteriaceae* (family level); Other, *Clostridium*, Clostridiales (order level), Bacteroides, and *Ruminococcus* for feed + cecal control group (Fig 6E). For the Calrose treatment group, Clostridiales (order level), *Oscillospira*, *Lachnospiraceae* (family level), *Lachnospiraceae* (family level); Other, and Streptophyta (order level) were commonly present as the major genera. The Calrose treatment group yielded a significantly lower abundance of *Enterobacteriaceae* (family level) with 0.28% (group average value) compared to the other control groups (25.59 and 36.88% for cecal only and feed + cecal control, respectively). Since the *Salmonella* belongs to the family *Enterobacteriaceae*, the significant decreases in relative abundance of *Enterobacteriaceae* by Calrose treatment determined by microbiome analysis are in accordance with the results showing a significant reduction in *Salmonella* populations by Calrose treatment determined by bacterial culture method.

### Metabolite profiles reveal small molecules and metabolic pathways with potential anti-*Salmonella* activity

**Fig 7:**
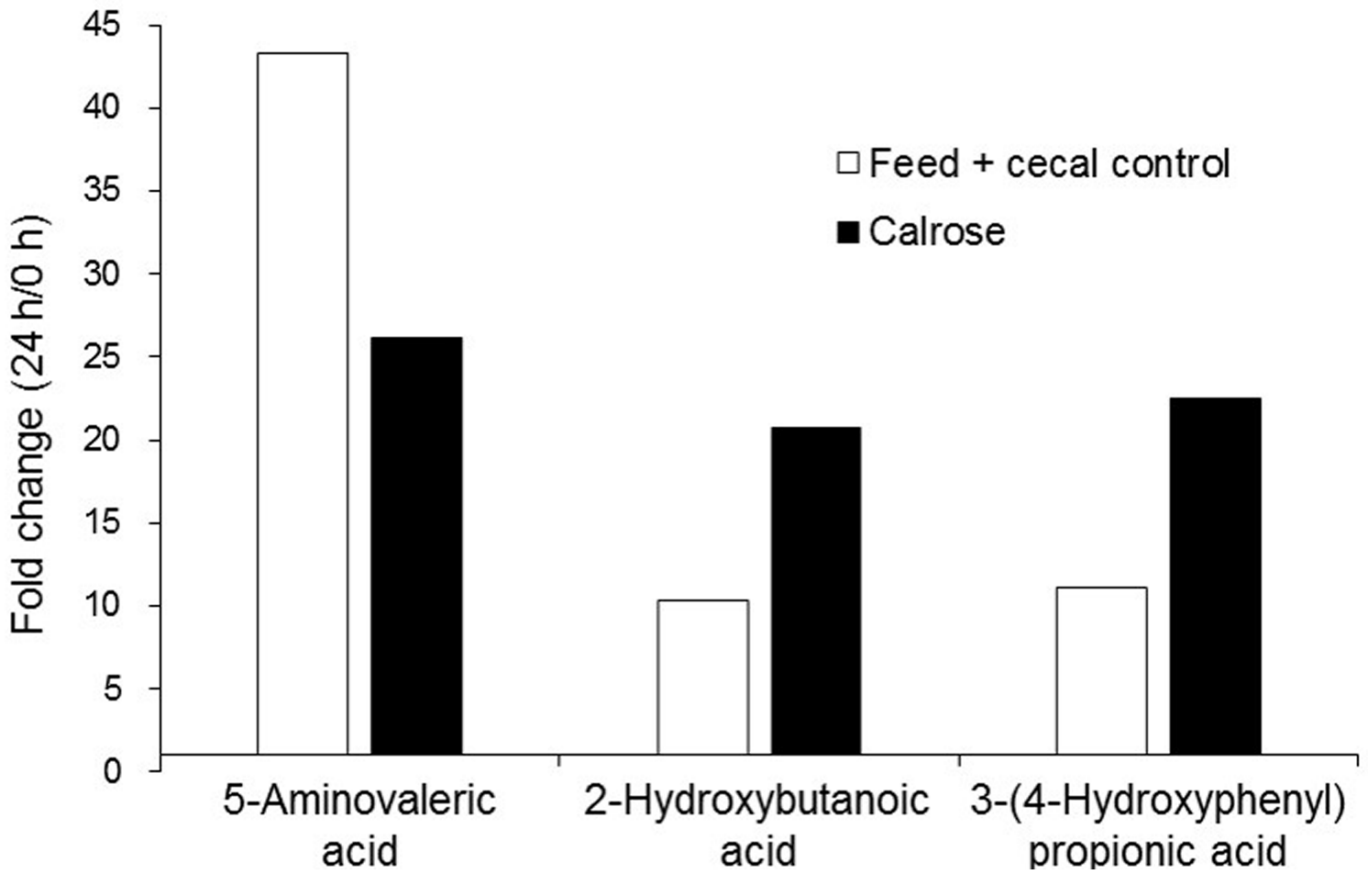
Metabolites that increased ≥ 10 fold over the course of the 24 h anaerobic culture, comparing the fold change (24 h/0 h) for feed + cecal control (negative control) and Calrose cultures.

**Fig 8:**
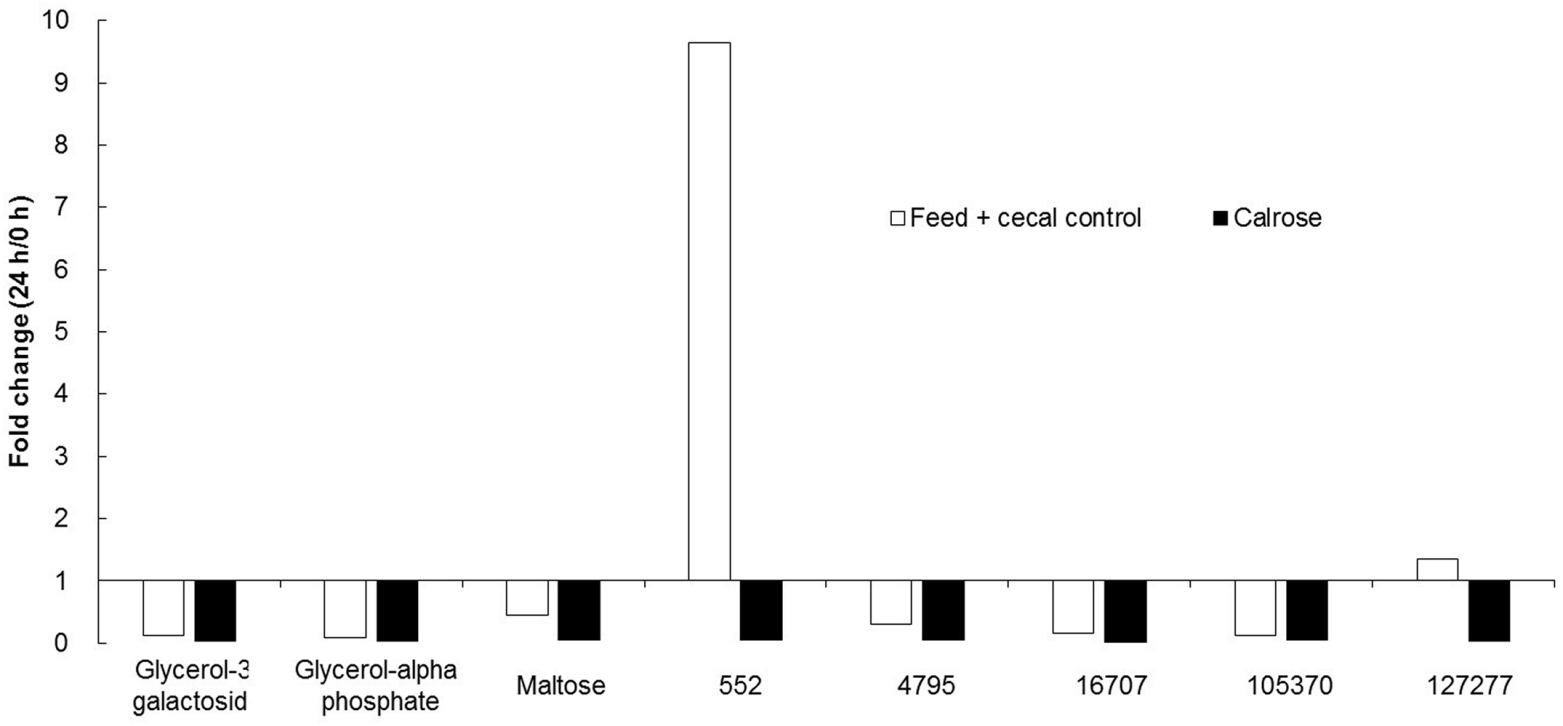
Metabolites that decreased ≥ 20 fold over the course of the 24 h anaerobic culture, comparing the fold change (24 h/0 h) for feed + cecal control (negative control) and Calrose cultures.

**Fig 9:**
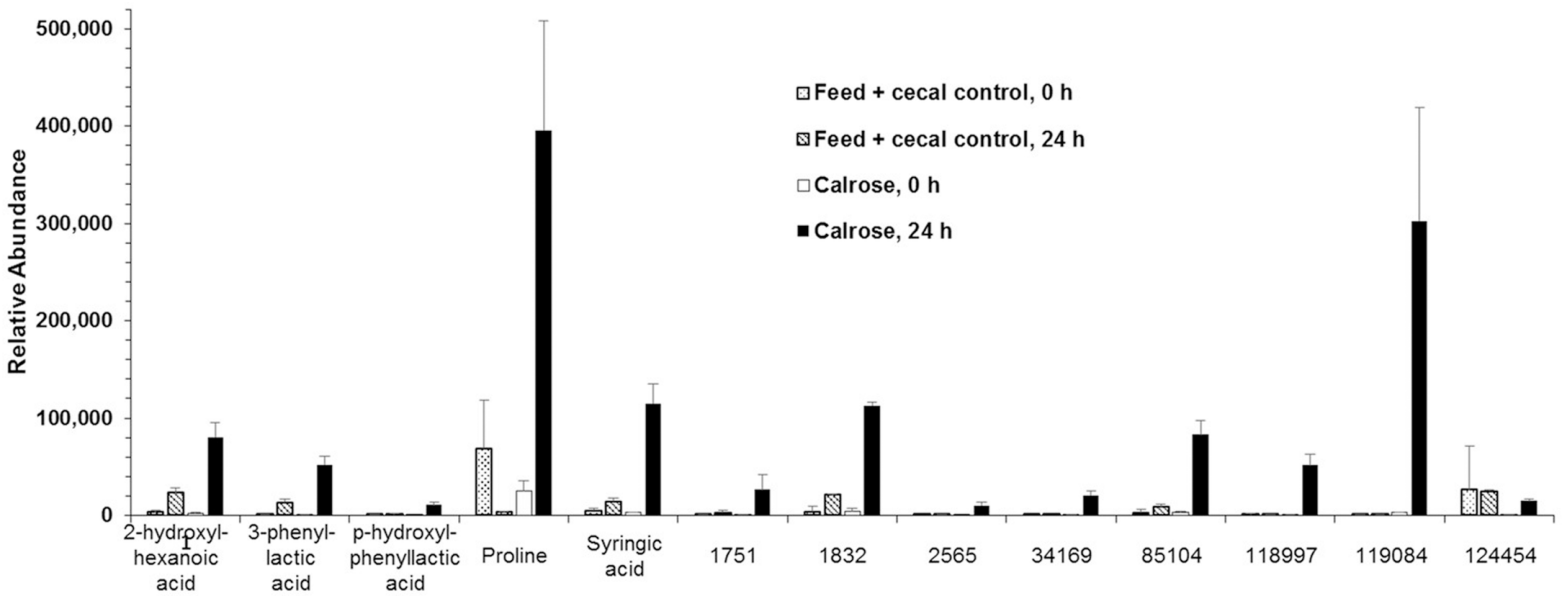
Metabolites that increased > 20 fold over the course of the 24 h Calrose anaerobic cultures, and the corresponding increases for the feed + cecal control (negative control) cultures.

Metabolite analysis was performed with only the Calrose supplemented incubations, since Calrose showed the most significant antimicrobial activity among the three cultivars of rice tested. Supernatants from the anaerobic cultures were collected at 0 and 24 h after initiating anaerobic incubation of the cecal contents with Calrose rice bran. Feed + cecal control cultures (negative control, NC) lacking rice bran were collected at the same time points. A total of 578 metabolites were detected, of which 211 were identified and 367 were unknown. A few metabolites related biochemically to short-chain fatty acids were increased in both NC and Calrose cultures (Fig 7). In addition, there were eight metabolites found to be reduced in Calrose cultures relative to the NC cultures (Fig 8). A 20-fold increase was chosen as an arbitrary cutoff for consideration of metabolites increased in the Calrose bran treatment. Those metabolites with a greater than 20 fold increase in the Calrose cultures and the corresponding levels in NC cultures are shown in Fig 9. Of these, a number of the metabolites were found to be statistically greater in abundance in the 24 h Calrose-containing cultures compared to the 24 h NC cultures (Table 2). Some of these metabolites can be explained as specific or predominant to rice, such as 1,2-anhydro-myo-inositol and inositol-4-monophosphate, which are components of phytate. Phytate, or phytic acid, is a highly substituted form of inositol, chemically designated as myo-inositol (1,2,3,4,5,6) hexakis phosphate, in which the hydroxyl groups have been esterified by addition of six phosphate groups. These phosphate groups chelate mineral cations, including calcium, and thus can reduce calcium uptake, leading to reduced bone minerals in chicks [30, 31]. The high phosphorus content of the phytate in rice bran has also been shown to increase the phosphorous content of chicken waste [30–32]. The reduction of calcium uptake results in bone deficiency [30], while the increased phosphate in manure emissions is detrimental to water quality and can contribute to eutrophication [32]. The presence of phytate in rice bran points out the value of differentiating between the components of rice bran that are beneficial and those such as phytate that could be detrimental. Thus, it will be necessary to separate the *Salmonella*-inhibiting component(s) from the phytate by chemical fractionation.

**Table 2:** Identified metabolites with >10-fold greater abundance in Calrose-treated 24 h anaerobic cultures compared to negative control cultures.

Metabolite Name	Mean Calrose RA*	Mean Neg. Control RA	Fold Difference (Calrose/Neg. control) **	*p-value*	Possible metabolic pathways
1,2-Anhydro-myo-inositol	3,939 ± 711	344 ± 283	11.4	0.0484	Phytate metabolism
3-Hydroxy-3-methylglutaric acid	6,254 ± 1,109	272 ± 9	23.0	0.0324	Branched-chain amino acid metabolism
Allantoic acid	8,087 ± 1,171	207 ± 12	39.0	0.0210	Glycine biosynthesis, xylene degradation
Conduritol beta-epoxide	85,416 ± 14,288	321 ± 80	266.4	0.0272	Inhibitor of beta-glucosidases, free-radical generation
Glutamic acid	145,554 ± 28,817	3,550 ± 813	41.0	0.0376	Glutathione metabolism, glyoxylate and dicarboxylate metabolism, arginine biosynthesis, alanine/aspartate/glutamate metabolism, carbapenem biosynthesis, histidine metabolism, ornithine metabolism
Hexitol	5,060 ± 981	137 ± 41	36.8	0.0348	Osmoprotection, osmoregulation
Inositol-4-monophosphate	12,256 ± 1,008	601 ± 448	20.4	0.0130	Inositol phosphate metabolism, phytate metabolism, sugar alcohol metabolism
Malonic acid	2,577 ± 75	156 ± 14	16.5	0.0012	Beta-alanine biosynthesis, fatty acid biosynthesis, fatty acid beta-oxidation
Methionine	39,817 ± 8,382	3,293 ± 1,172	12.1	0.0423	Ethylene biosynthesis, methanethiol biosynthesis, homocysteine metabolism, 1-carbon metabolism via conversion into S-adenosyl methionine
Ornithine	438,156 ± 71,171	28,958 ± 2,578	15.1	0.0309	Arginine catabolism, butyric acid synthesis, urea cycle
Pantothenic acid	2,594 ± 203	182 ± 49	14.3	0.0069	Fatty acid and B-vitamin metabolism
Phosphogluconic acid	21,927 ± 2,018	1192 ± 947	18.4	0.0152	Purine, pyrimidine, and histidine metabolism
Pinitol	297,330 ± 49,993	223 ± 58	1335.3	0.0271	Inositol metabolism, inositolphosphoglycan metabolism
Vanillic acid	7,071 ± 806	491 ± 126	14.4	0.0105	Aminobenzoate degradation
Xylitol	7,039 ± 1448	482 ± 37	14.6	0.0476	Pentose sugar metabolism

* RA, relative abundance ± standard error

** Fold difference was determined by dividing the relative abundance of Calrose by that of the Negative Control.

Other known metabolites found that may be rice-specific are conduritol beta-epoxide, hexitol, and pinitol. These possibly represent an inhibitor of beta-glucosidases and osmoprotectants, respectively. Numerous plant organs express beta-glucosidases during development, and inhibitors may be required to regulate their activity when they are no longer required as part of specific developmental events [33]. Osmoprotectants are compatible solutes that accumulate within cells during periods of osmotic stress caused by high osmolarity (high solute concentration) of the environmental surroundings. This accumulation relieves the osmotic stress while avoiding passive, and often toxic, volume depletion within the cell in response to the high osmolarity [34].

Of particular interest to the anti-*Salmonella* properties of rice bran is the increased abundance of malonic acid, ornithine, and pantothenic acid in Calrose cultures relative to the control cultures (Table 2). These metabolites may be functioning in fatty acid synthesis, and catabolism [35]. Previous studies have indicated that short-chain fatty acids (SCFA) can inhibit *Salmonella* through a mechanism of decoupling of ATP formation through membrane damage or by denaturation of acid-sensitive proteins [36]. Interestingly, butyric acid has also been observed to down-regulate pathogenicity island gene expression [37], suggesting that fatty acids can reduce the virulence of *Salmonella*. Thus an increase in fatty acid synthesis in 24 h anaerobic cultures may have an inhibitory effect on *Salmonella* systemic infection by restricting host invasion or other functions. This can be further explored in live bird *Salmonella* challenge studies where organ invasion could be examined.

A small number of fatty acid metabolites were increased in both Calrose and negative control cultures after 24 h of anaerobic growth compared to the 0 h culture supernatants (Fig 8). These appear consistent with the hypothesis that the anaerobic culturing promoted differences in fermentation. A potentially interesting metabolite of unknown structure, designated 552, was considerably reduced in Calrose cultures compared to control cultures at 24 h (Fig 9).

In conclusion, the cultures containing Calrose bran had significantly less *Salmonella* recovered compared to control cultures without rice bran. Our results indicate that bran from Calrose rice is more effective at inhibiting *S*. Typhimurium in comparison to the two other rice brans tested, and this may be due in part to its effects on the metabolic profile. In addition, the microbiome results of this study obtained from *in vitro* poultry cecal incubations indicate that shifts in the cecal microbiota composition as well as metabolic and fermentation activities may also contribute to the reduction *S*. Typhimurium. One implication of these experiments is that Calrose rice bran or an extracted component thereof could have applications in poultry production as a feed component to reduce dependence on antibiotics, improving the microbiological safety of chicken products. Chemical fractionation experiments to determine the specific biochemical component(s) responsible for the *Salmonella*-inhibitory effect observed also appear worthwhile. To achieve this, future feeding and challenge studies need to be conducted to examine the *in vivo* responses, including possible effects on bone density, immune response modulation, and the environmental implications of feeding rice bran. In addition, other foodborne disease-causing serovars, such as *S*. Enteritidis and *S*. Heidelberg, should be examined to gauge the spectrum of antimicrobial activity possessed by Calrose. Other factors needing more attention are the possible effects of Calrose bran on microbiome changes that in turn affect *Salmonella* colonization. Taken together, our data suggest that Calrose rice bran warrants further study as a practical prebiotic additive in poultry feed to limit cecal colonization with *Salmonella*.
